# Nano‐Mediated Fluorescence Switching for Epidermal Growth Factor Receptor Detection

**DOI:** 10.1111/cpr.70063

**Published:** 2025-05-14

**Authors:** Xin Fu, Yuhao Wang, Wenxin Zhang, Yuepeng Yang, Jialin Zeng, Xiaodie Li, Chengyu Feng, Bin Li, Yingying Liu, Yinan Zhang, Chao Zhang, Sicong Ma

**Affiliations:** ^1^ Department of Oncology, Zhujiang Hospital Southern Medical University Guangzhou China; ^2^ School of Inspection Ningxia Medical University Yinchuan China; ^3^ Department of Pediatric Hematology, Zhujiang Hospital Southern Medical University Guangzhou China; ^4^ School of Chemistry and Chemical Engineering, Center for Transformative Molecules, Zhangjiang Institute for Advanced Study and National Center for Translational Medicine (Shanghai) Shanghai Jiao Tong University Shanghai China; ^5^ School of Chemical Science and Engineering Tongji University Shanghai China; ^6^ Department of Intensive Care Medicine, Zhujiang Hospital Southern Medical University Guangzhou China; ^7^ Global Health Research Center, Guangdong Provincial People's Hospital Guangdong Academy of Medical Sciences Guangzhou China

**Keywords:** aptamer, black phosphorus nanosheets (BPNSs), cancer diagnostics, epidermal growth factor receptor (EGFR)

## Abstract

Identification of the epidermal growth factor receptor (EGFR) in biological specimens is essential for cancer diagnostics, drug development and therapeutic monitoring. However, real‐time techniques for accurate EGFR expression monitoring are currently limited. In this study, we report the development of a novel nano detector (Cy3‐Apt_EGFR_@BPNSs) with the capabilities of quenching and recovery to enable visual EGFR expression analysis. Cy3‐Apt_EGFR_ is a Cy3‐labelled single‐stranded RNA (ssRNA) that exhibits specific binding to EGFR. Black phosphorus nanosheets (BPNSs) possess the ability to adsorb Cy3‐Apt_EGFR_ via van der Waals forces, quenching its fluorescence when combined. The detection of EGFR receptors on cancer cell surfaces prompts the release of Cy3‐Apt_EGFR_ from BPNSs, a consequence of the robust binding interaction between the receptor and aptamer, thereby leading to fluorescence reinstatement. The recovered fluorescence intensity of this detector is found to be directly correlated with EGFR expression levels in cancer cells, indicating its potential for guiding tumour diagnosis and treatment. The specificity of Cy3‐Apt_EGFR_@BPNSs further enhances its utility in detecting EGFR. More importantly, our research demonstrates that the reduction in EGFR expression levels within cancer cells corresponds to a proportional decline in fluorescence intensity, thereby facilitating precise tracking of EGFR dynamics.

## Graphical abstract

This study presents Cy3‐Apt_EGFR_@BPNSs, a novel nano‐detector for real‐time, visual monitoring of EGFR expression in cancer cells. Addressing the limitations of current methods, this detector utilises fluorescence quenching/recovery, providing high specificity and sensitivity, with fluorescence intensity proportional to EGFR levels. It offers potential for early diagnosis, personalised treatment, and therapeutic monitoring in cancer.

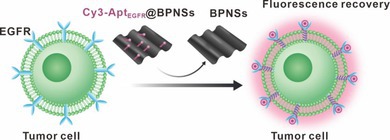

## 1 Introduction

The epidermal growth factor receptor (EGFR), which belongs to the four‐member ErbB family of tyrosine kinase growth factor receptors, serves as a transmembrane glycoprotein situated on the cell surface. It gets activated upon the binding of several growth factors, such as epidermal growth factor and transforming growth factor [1, 2, 3]. Activation and autophosphorylation of EGFR initiate a cascade of intracellular signalling pathways, which govern critical cellular functions including cell proliferation, adhesion, migration and apoptosis [4, 5, 6]. Epithelial tissue development and homeostasis are intricately regulated in vivo by EGFR. The EGFR gene is one of the most frequently mutated genes in a variety of cancers, such as gastroesophageal adenocarcinoma, lung cancer, breast cancer, carcinoma of the bladder, rectal cancer and glioblastoma [7, 8, 9]. Moreover, EGFR mutations are associated with advanced disease stages and the development of resistance to conventional treatment modalities like chemotherapy and radiation therapy [10, 11]. Although other related proteins, such as HER2 and VEGF, could be considered, the extensive research and established clinical applications of EGFR make it a compelling target for investigation [12, 13]. Therefore, EGFR represents a target for antitumor strategies and gene therapies. Consequently, assessing EGFR expression levels, particularly in cases of overexpression, has gained increasing attention as a valuable tool for cancer screening and treatment management [14, 15, 16].

Conventional methodologies for the detection of EGFR encompass enzyme‐linked immunosorbent assay (ELISA), Western blotting (WB) and tissue immunohistochemistry (IHC) [17, 18, 19, 20]. However, these techniques are rather time‐intensive, since they entail multiple sequential steps and the utilisation of chromophores, fluorophores, or enzymatic labelling agents. Additionally, both IHC and WB demand highly proficient personnel for the proper execution of the procedure [21, 22, 23]. Consequently, there exists an urgent need to develop a straightforward, efficient, and direct method for the detection of EGFR.

Black phosphorus nanosheets (BPNSs) are commonly used fluorescence quenching nanomaterials [24, 25]. Compared to other fluorescence quenching nanomaterials such as metal nanoparticles, graphene and MoS_2_, BPNSs have a layered structure similar to graphene, with a high specific surface area and active sites. Additionally, BPNSs offer advantages such as excellent optical properties, high carrier mobility, good biocompatibility and easy functionalization [26, 27, 28, 29, 30, 31].

**SCHEME 1:**
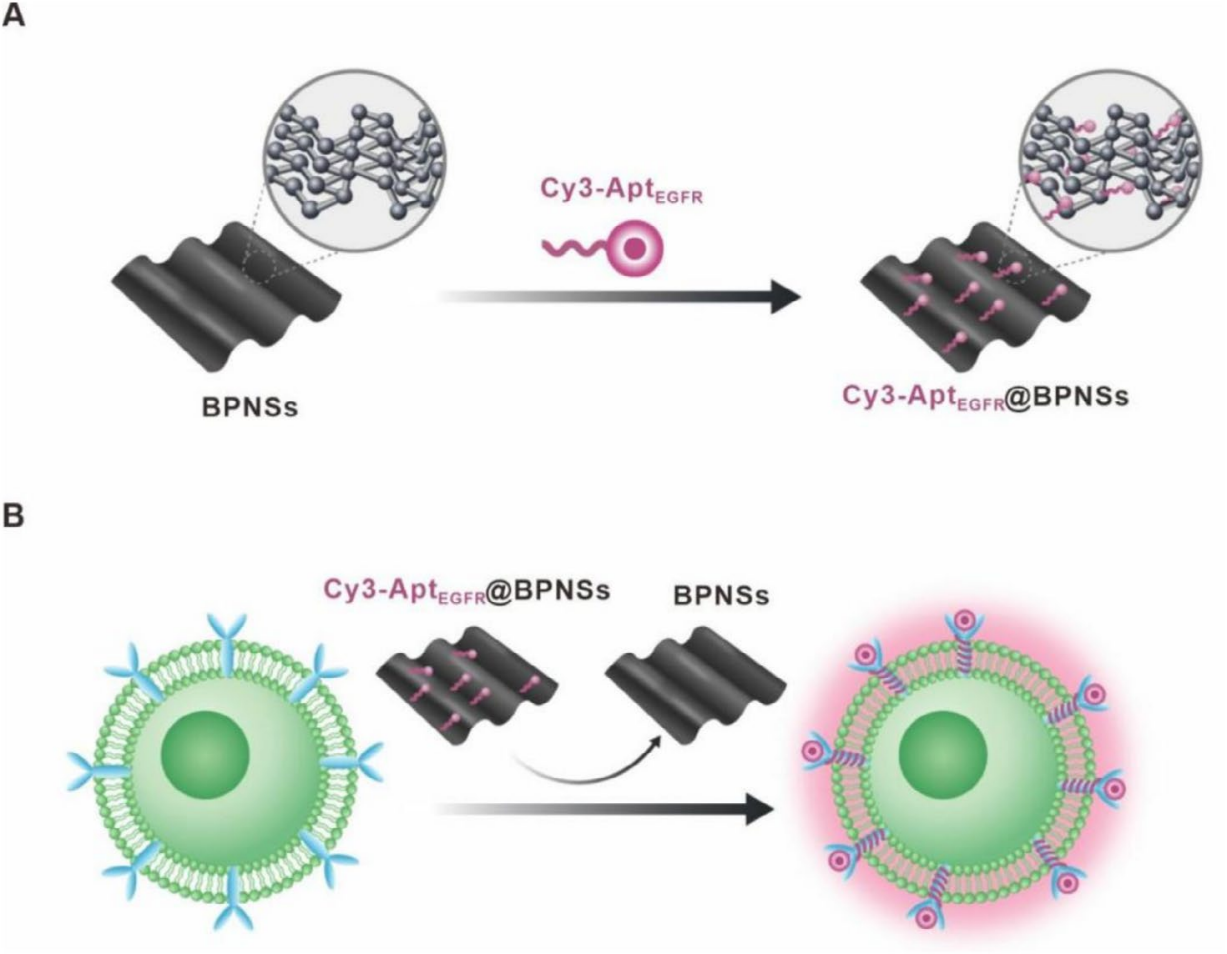
Illustration depicting the principle of Cy3‐Apt_EGFR_@BPNSs. (A) Schematic diagram of Cy3‐Apt_EGFR_ loaded onto BPNSs. The Cy3‐Apt_EGFR_ is quenched when bound to BPNSs. (B) Functional diagram of Cy3‐Apt_EGFR_@BPNSs, where fluorescence intensity indicates the levels of EGFR expression.

In this study, we present an inventive strategy for the visualisation and detection of EGFR expression by creating a nano‐enabled quenching and recovery detector on the basis of multilayered BPNSs functionalized with Cy3‐labelled EGFR aptamers (Cy3‐Apt_EGFR_@BPNSs) [32, 33, 34]. Previous studies have demonstrated that BPNSs can adsorb and interact with dye‐labelled ss‐DNA (or RNA) aptamers through van der Waals forces between the nucleobases and the phosphoenoligomer surfaces, resulting in fluorescence quenching [35, 36, 37, 38]. As depicted in Scheme 1A, the fluorescence of Cy3‐labelled aptamer targeting the EGFR (Cy3‐Apt_EGFR_) is effectively quenched upon its interaction with BPNSs. This quenching phenomenon occurs primarily due to the van der Waals forces that facilitate the binding between the Cy3‐Apt_EGFR_ and the BPNSs. Upon the subsequent recognition of the EGFR receptor present on the surface of cancer cells, the Cy3‐Apt_EGFR_ is released from the BPNSs. This release is attributed to the high affinity interaction between the EGFR receptor and the aptamer, which is specifically designed to bind to this receptor with high specificity and strength. As a result of this disassociation, the fluorescence signal of Cy3‐Apt_EGFR_ is restored, as illustrated in Scheme 1B. This fluorescence recovery serves as a significant indicator of the presence of EGFR on the cancer cell surface, highlighting the potential of this system for targeted cancer diagnostics and therapeutic applications. Consequently, fluorescence intensity correlates positively with EGFR expression, providing a quantitative readout of EGFR levels in targeted cells. Additionally, the specificity of Cy3‐Apt_EGFR_@BPNSs in detecting EGFR has also been demonstrated. More importantly, the downregulation of EGFR expression levels in cancer cells correspondingly decreases the fluorescence intensity of Cy3‐Apt_EGFR_@BPNSs, facilitating real‐time monitoring of EGFR dynamics. We anticipate that Cy3‐Apt_EGFR_@BPNSs can intuitively and dynamically monitor EGFR expression levels in tumours, providing a robust imaging framework to guide the adoption of treatments and improve prognosis.

## 2 Results and Discussion

### 2.1 Synthesis and Characteristics of Cy3‐Apt_EGFR_@BPNSs

**FIGURE 1:**
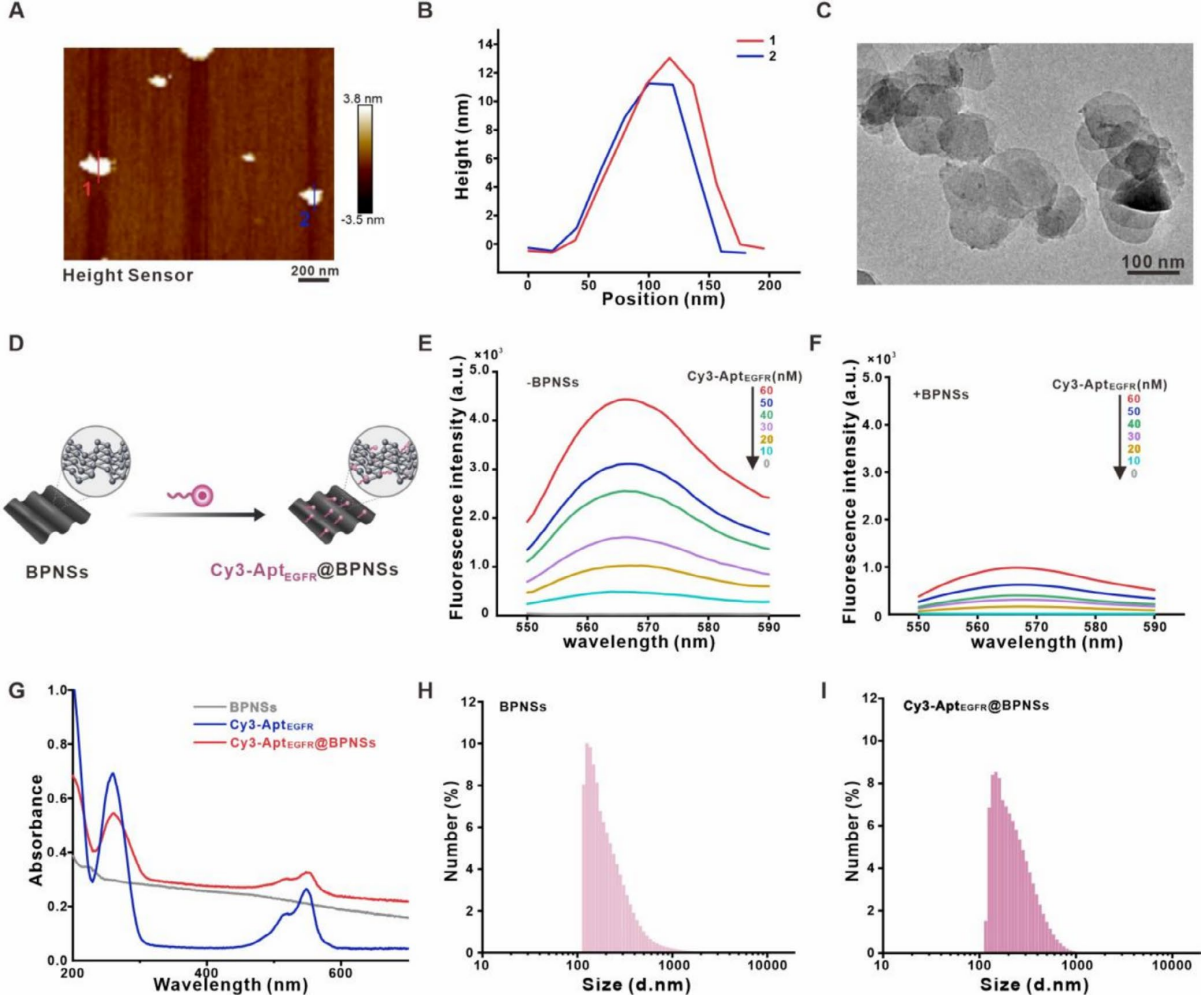
Characteristics of Cy3‐Apt_EGFR_@BPNSs. (A) Representative AFM image of randomly selected samples of BPNSs. (B) Height distribution of the lines in (A). (C) TEM image of randomly selected samples of BPNSs. (D) Schematic representation of Cy3‐Apt_EGFR_ synthesis with BPNSs. (E, F) Fluorescence spectra of Cy3‐Apt_EGFR_, assessed across a concentration gradient both without and with BPNSs integration. (G) UV–Vis absorption profiles for BPNSs, Cy3‐Apt_EGFR_, and Cy3‐Apt_EGFR_@BPNSs. (H, I) Hydrodynamic diameter profiles of BPNSs and Cy3‐Apt_EGFR_@BPNSs.

BPNSs, which constitute a particular subclass within the family of two‐dimensional materials, are organised in a hexagonal lattice structure formed by a monolayer of black phosphorus atoms. To characterise the morphology as well as the size of the BPNSs, atomic force microscopy (AFM) and transmission electron microscopy (TEM) techniques were employed. As shown in Figure 1A,C, the BPNSs exhibit overlapping folds of varying thicknesses, with heights of 13.1 and 11.3 nm denoted as lines 1 and 2, respectively (Figure 1B). The TEM images further indicate that the nanosheets are approximately 120 nm in length (Figure 1C). The ratio of Cy3‐Apt_EGFR_ to BPNSs was examined using agarose gel electrophoresis to determine the optimal loading concentration of Cy3‐Apt_EGFR_. Gel imaging and quantitative analysis revealed that at a BPNSs concentration of 0.2 mg/mL, free DNA signals were absent when the Cy3‐Apt_EGFR_ concentration fell below 40 nM, indicating that the Cy3‐Apt_EGFR_ was nearly completely loaded onto the BPNSs (Figures S1 and S2).

When Cy3‐Apt_EGFR_ is adsorbed on the surface of BPNSs, BPNSs efficiently quench the fluorescence of Cy3‐Apt_EGFR_ (Figure 1D). To verify this, the fluorescence characteristics of Cy3‐Apt_EGFR_ were first explored in the absence of BPNSs. With an increase in the concentration of Cy3‐Apt_EGFR_, the fluorescence intensity was significantly enhanced, with the characteristic emission spectrum peaking within 565–575 nm (Figure 1E). Subsequently, in the presence of BPNSs, there was a significant attenuation in the fluorescence intensity of Cy3‐Apt_EGFR_ (Figure 1F). It is crucial to emphasise that the adsorption process of Cy3‐Apt_EGFR_ onto BPNSs manifests the most prominent quenching effect when the concentrations are lower than 40 nM. This further validates and confirms that Cy3‐Apt_EGFR_ at concentrations beneath 40 nM can be efficiently loaded onto BPNSs.

In addition, the results of ultraviolet–visible absorption spectroscopy analysis demonstrated that Cy3‐Apt_EGFR_ exhibited a characteristic absorption at a wavelength of 260 nm. This characteristic absorption peak at 260 nm was preserved in Cy3‐Apt_EGFR_@BPNSs, thus confirming the successful fabrication of Cy3‐Apt_EGFR_@BPNSs (Figure 1G). The successful synthesis of Cy3‐Apt_EGFR_@BPNSs was further corroborated by the dynamic light scattering (DLS) analysis (Figure 1H,I). Cy3‐Apt_EGFR_@BPNSs exhibited a mean hydrodynamic diameter of around 148, 12 nm larger than the bare BPNSs. To assess the stability of the synthesised Cy3‐Apt_EGFR_@BPNSs, filtrates were collected at various time points and analysed at room temperature. The CCK‐8 results showed that the Cy3‐Apt_EGFR_@BPNSs also have good biosafety (Figure S3). The absorption peak of Cy3‐Apt_EGFR_ was absent from all groups, suggesting that Cy3‐Apt_EGFR_ did not dissociate from Cy3‐Apt_EGFR_@BPNSs (Figure S4). These results indicate the successful synthesis of Cy3‐Apt_EGFR_@BPNSs and demonstrate its superior fluorescence quenching properties and stability.

### 2.2 The Responsiveness of Cy3‐Apt_EGFR_@BPNSs to EGFR

**FIGURE 2:**
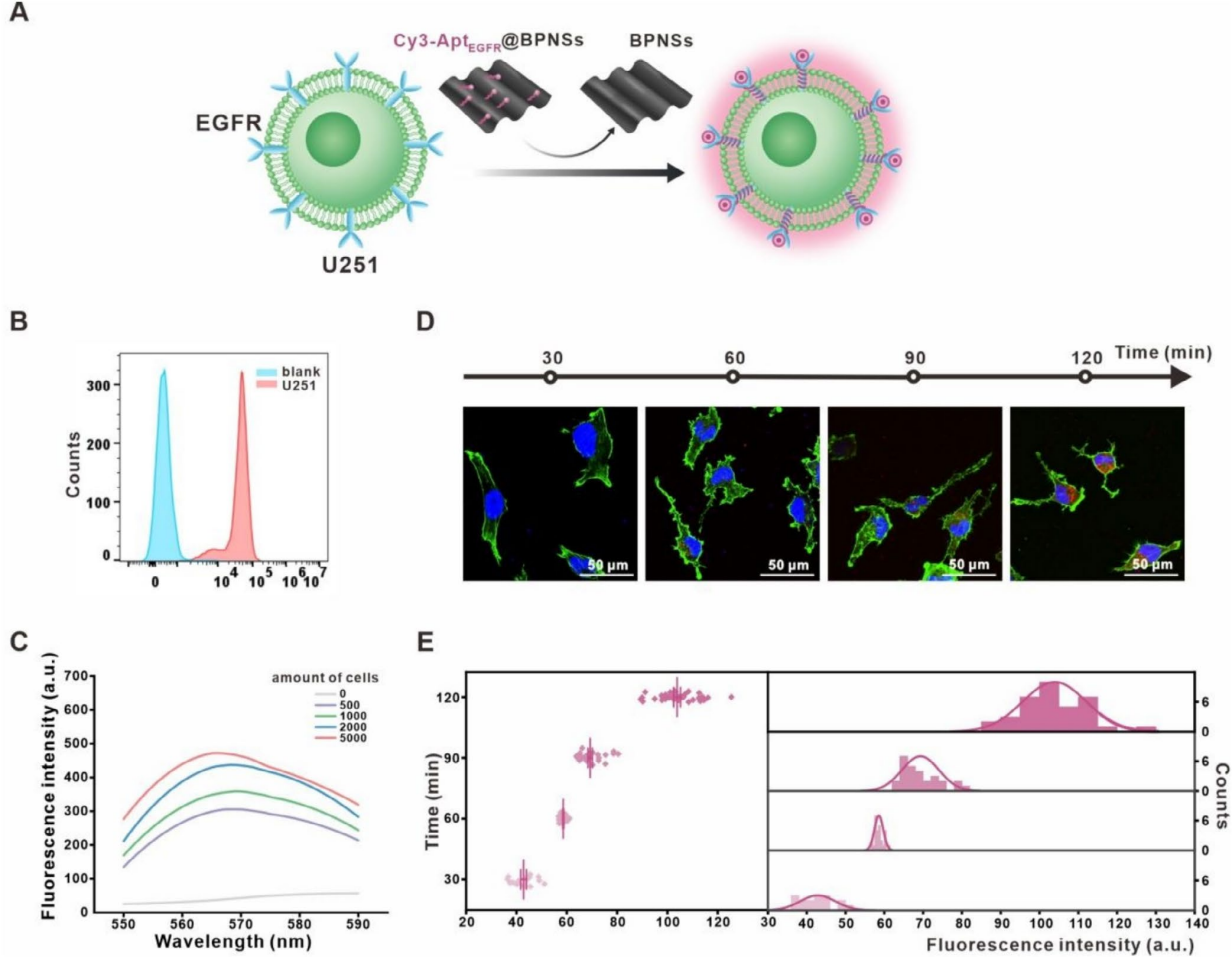
Assessment of Cy3‐Apt_EGFR_@BPNSs' responsiveness to EGFR expression in U251 Cell Line. (A) Diagram showing EGFR detected in U251 cells. (B) Flow cytometry analysis of antibody fluorescence in U251 cells. (C) Fluorescence intensity of different amounts of U251 cells. (D) LSCM images of cells following treatment with Cy3‐Apt_EGFR_@BPNSs across varied incubation durations. (E) Analysis of fluorescence intensity data from (D).

Based on previous studies indicating that EGFR amplification occurs in 50% of primary gliomas, we selected two glioma cell lines, U251 and U87MG, to validate the responsiveness of Cy3‐Apt_EGFR_@BPNSs to EGFR. Initially, we focused on U251 cells for in vitro experiments (Figure 2A). We conducted flow cytometric quantification of EGFR protein expression using fluorescently labelled antibodies, confirming substantial overexpression of EGFR on the surfaces of U251 cells (Figure 2B). Subsequently, fluorescence recovery experiments were carried out with varying cell densities on U251 cells, demonstrating that fluorescence intensity increased with cell number, indicating a positive correlation between EGFR levels and fluorescence intensity (Figure 2C). The next step involved incubating U251 cells with Cy3‐Apt_EGFR_@BPNSs without light for different durations, after which fluorescence recovery was observed using a confocal microscope (Figure 2D). Statistical analysis was conducted to quantify the observed fluorescence (Figure 2E). The results indicated that fluorescence intensity increased with extended incubation time.

**FIGURE 3:**
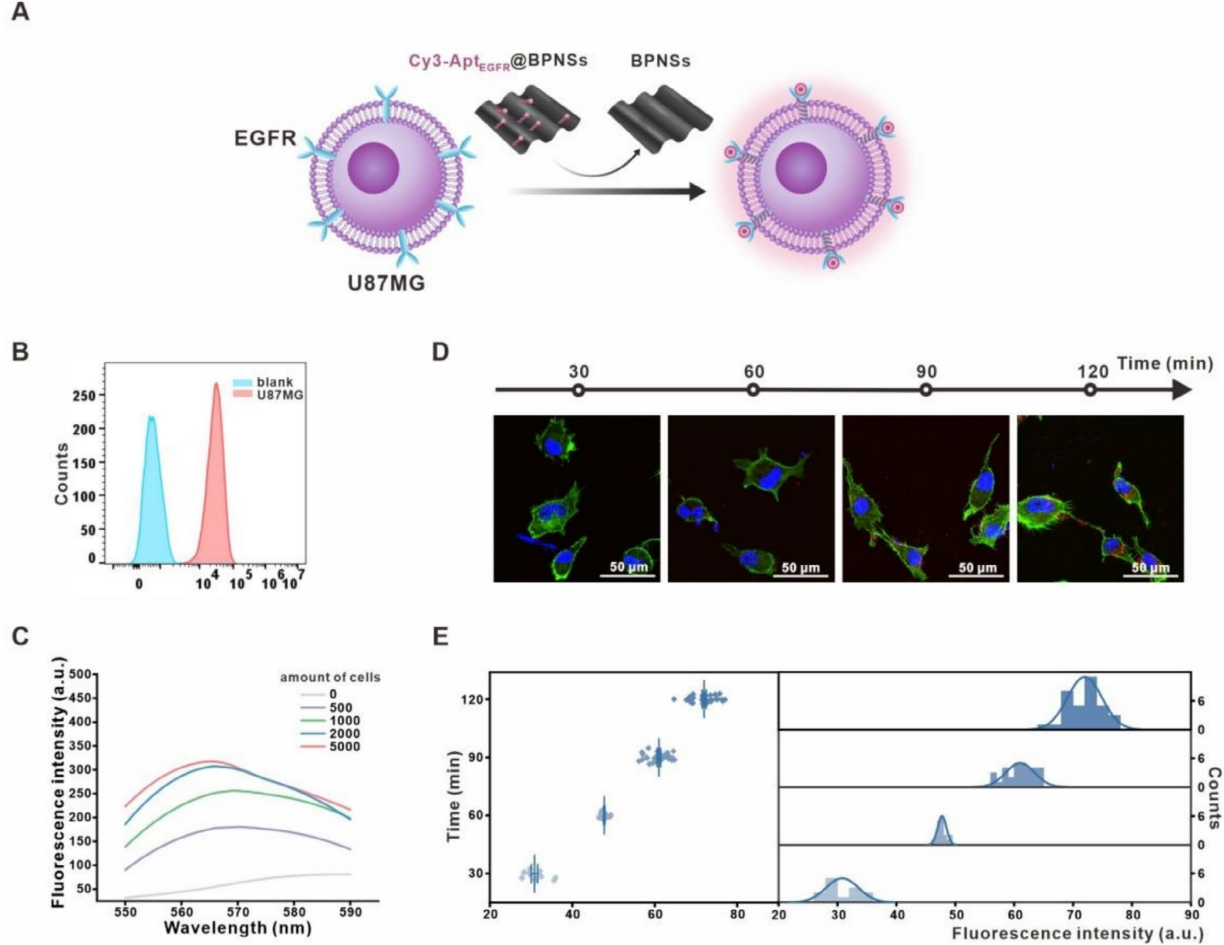
The response of Cy3‐Apt_EGFR_@BPNSs to EGFR expression in U87MG cell line. (A) Diagram showing EGFR detected in U87MG cells. (B) Flow cytometry analysis of antibody fluorescence in U87MG cells. (C) Fluorescence intensity of different amounts of U87MG cells. (D) LSCM images of cells following treatment with Cy3‐Apt_EGFR_@BPNSs across varied incubation durations. (E) Analysis of fluorescence intensity data from (D).

Next, we selected U87MG cells for further validation of the responsiveness of Cy3‐Apt_EGFR_ to EGFR (Figure 3A). EGFR expression on U87MG cells was quantified by flow cytometry using fluorescently labelled antibodies (Figure 3B). EGFR expression was found to be marginally decreased in U87MG cells relative to U251 cells, as evidenced by statistical analysis (Figure S5). Fluorescence recovery experiments conducted with U87MG cells at different cell densities (Figure 3C) and varying incubation times (Figure 3D,E) also demonstrated that fluorescence recovery in U87MG cells is proportional to both cell densities and incubation time. These results underscore the viability of Cy3‐Apt_EGFR_ as an EGFR detector.

### 2.3 The Capability of Cy3‐Apt_EGFR_@BPNSs for Specific Monitoring of EGFR

**FIGURE 4:**
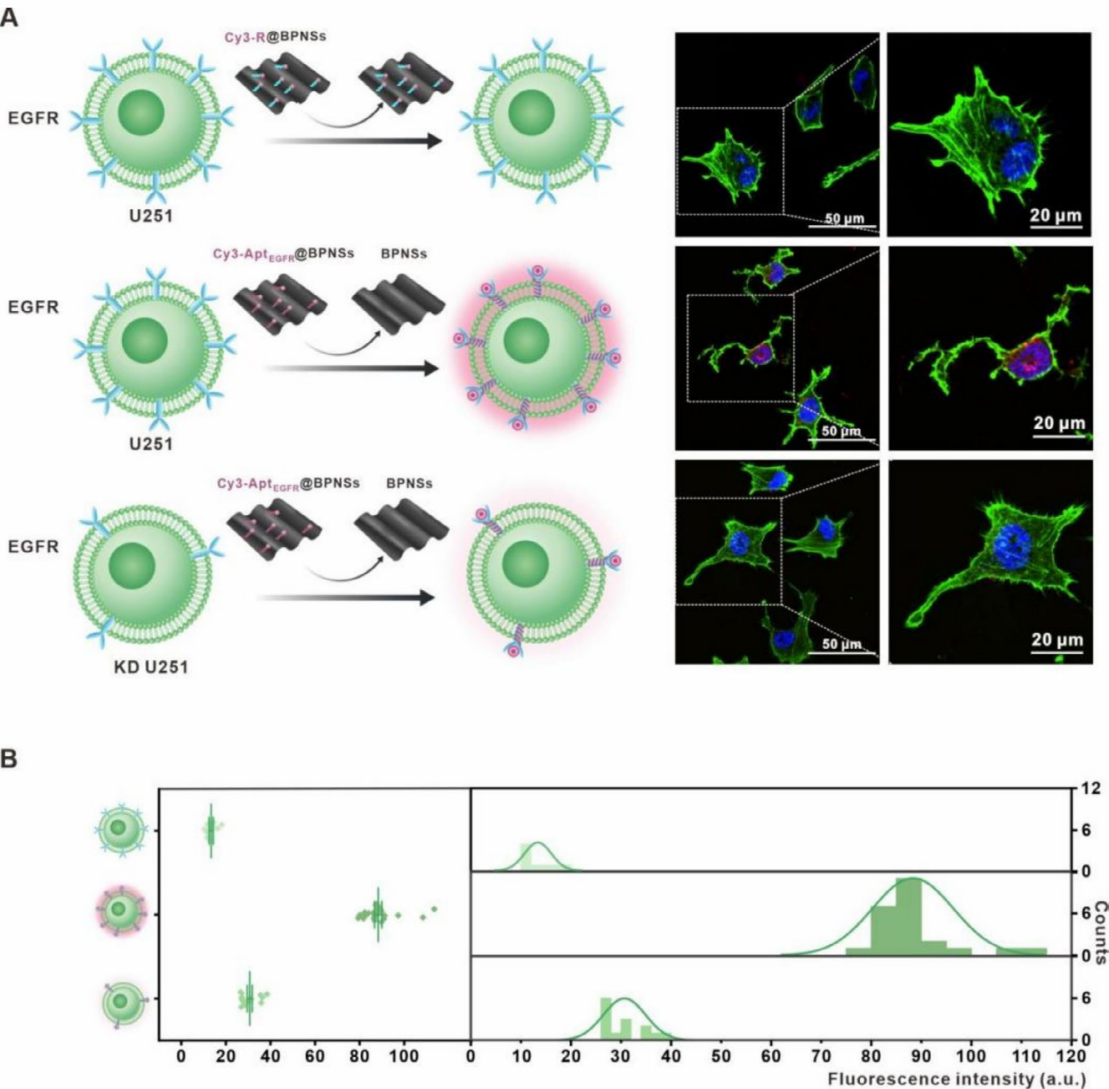
The Capability of Cy3‐Apt_EGFR_@BPNSs for specific monitoring of EGFR in U251 cell line. (A) Diagram and LSCM images showing EGFR detected in U251 sublines. (B) Data analysis of fluorescence intensity from (A).

To verify the specificity of Apt_EGFR_, a random single‐stranded RNA (ssRNA) sequence with the same number of nucleotides was generated using a small program called OligoCalculator. This sequence was employed to replace Apt_EGFR_, resulting in the formation of a new detector (Cy3‐R@BPNSs). As shown in Figure 4A, no significant fluorescence recovery was detected after incubating Cy3‐R@BPNSs with U251 cells, suggesting the necessity of Apt_EGFR_ for specific EGFR detection. Subsequently, we knocked down EGFR expression in U251 cells and incubated these modified cells with Cy3‐Apt_EGFR_@BPNSs. Similarly, no significant fluorescence recovery was observed, further validating the specificity of Cy3‐Apt_EGFR_@BPNSs for EGFR. The statistical results also confirmed the specificity of Cy3‐Apt_EGFR_@BPNSs for recognising the EGFR, thereby ruling out the possibility that the experimental results were influenced by non‐specific binding (Figure 4B).

### 2.4 The Capability of Cy3‐Apt_EGFR_@BPNSs for Accurate Monitoring of EGFR Dynamics

**FIGURE 5:**
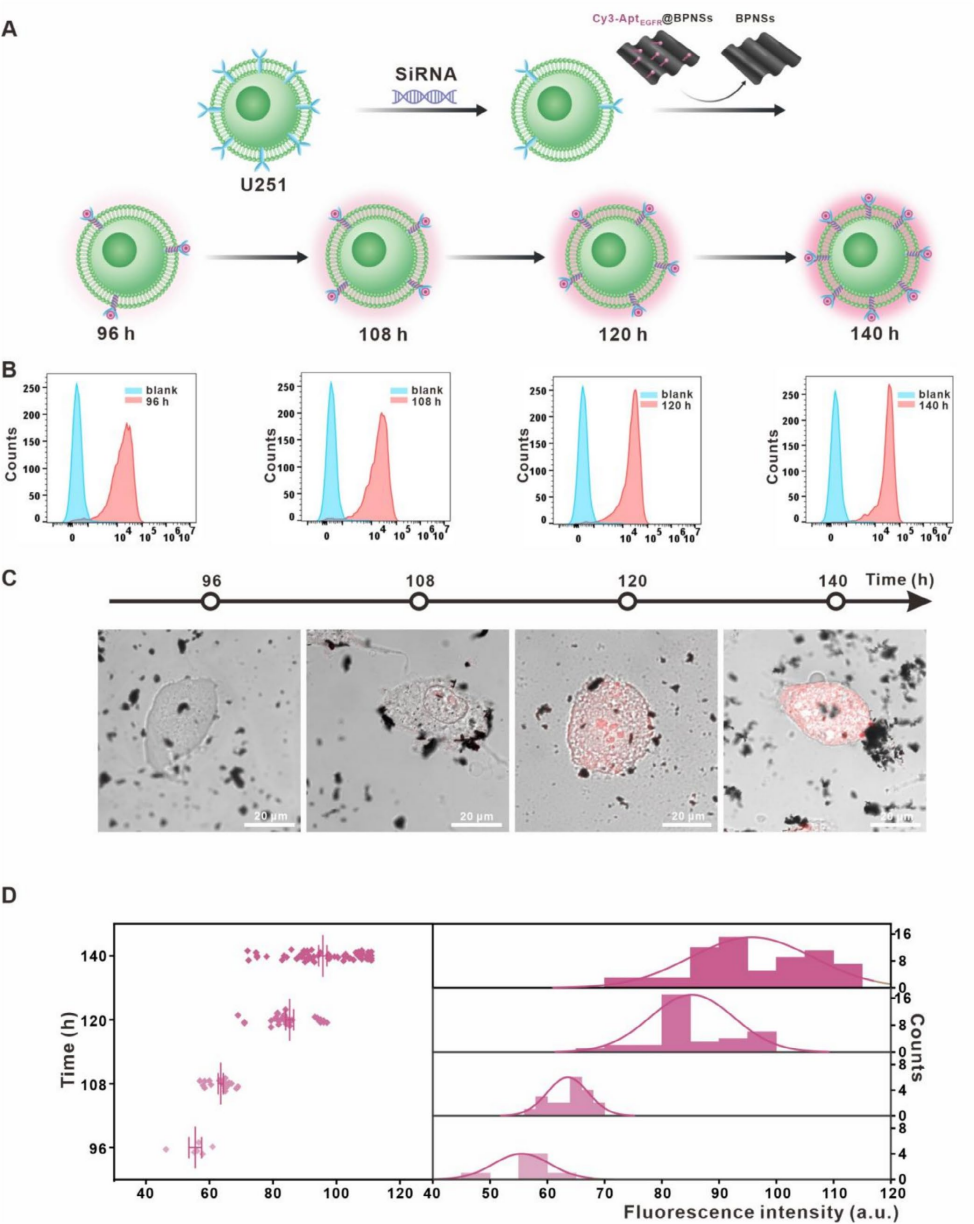
The Capability of Cy3‐Apt_EGFR_@BPNSs for accurate monitoring of EGFR dynamics in U251 cell line. (A) Schematic of EGFR detection following EGFR downregulation and recovery in U251 cells. (B) Flow cytometry analysis of antibody fluorescence in post‐transfection U251 cells at various time points (96, 108, 120, 140 h). (C) LSCM of Cy3‐Apt_EGFR_@BPNSs‐treated post‐transfection U251 cells at different time intervals (96, 108, 120, 140 h). (D) Statistical analysis of fluorescence intensity from (C).

To assess the effectiveness of Cy3‐Apt_EGFR_@BPNSs in monitoring EGFR dynamics in glioma cells (Figure 5A), we transfected small interfering RNA (siRNA) into U251 cells to knock down EGFR levels. Notably, flow cytometry analysis confirmed a progressive elevation in EGFR protein expression on the surface of U251 cells at 96, 108, 120 and 140 h post‐transfection (Figure 5B). The statistical analysis corresponding to the flow cytometry results can be found in Figure S6. This finding indicates that the knockdown method effectively achieves the dynamic changes in EGFR. To assess whether fluorescence recovery could monitor these dynamic changes, we incubated Cy3‐Apt_EGFR_@BPNSs with the modified U251 cells 72 h after the initial transfection. Changes in fluorescence intensity were continuously monitored using confocal microscopy, and the results indicated that fluorescence intensity increased over time (Figure 5C). Statistical analysis revealed that fluorescence recovery at 140 h exceeded three times that observed at 96 h (Figure 5D). These results strongly demonstrate the capability of Cy3‐Apt_EGFR_@BPNSs to detect EGFR dynamics in U251 cells.

**FIGURE 6:**
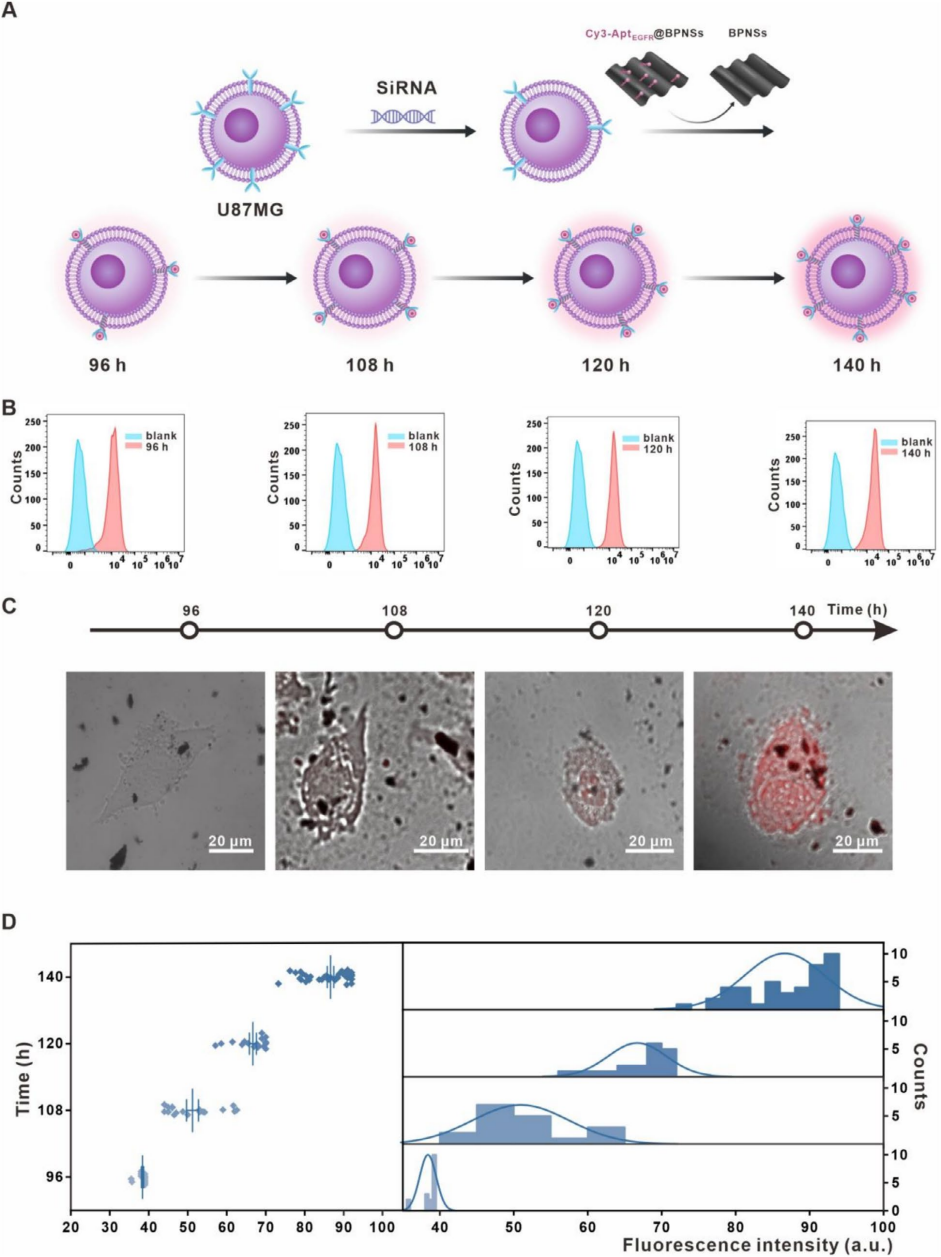
Dynamic detection to U87MG cell line. (A) Schematic of EGFR detection following EGFR downregulation and recovery in U87MG cells. (B) Flow cytometry analysis of antibody fluorescence in post‐transfection U87MG cells at various time points (96, 108, 120, 140 h). (C) LSCM of Cy3‐Apt_EGFR_@BPNSs‐treated post‐transfection U87MG cells at different time intervals (96,108,120,140 h). (D) Statistical analysis of fluorescence intensity from (C).

To further evaluate the applicability of the dynamic monitoring method, we investigated the responsiveness of Cy3‐Apt_EGFR_@BPNSs in another glioma cell line, U87MG (Figure 6A). We repeated the transfection process in U87MG cells to reduce EGFR expression levels and observed a similar trend, with EGFR protein expression on the surface of U87MG cells gradually increasing at 96, 108, 120 and 140 h post‐transfection (Figure 6B and Figure S7). This consistent result corroborates the efficacy of the knockdown method in capturing dynamic EGFR changes across different glioma cell types. Subsequently, we incubated Cy3‐Apt_EGFR_@BPNSs with U87MG cells and observed a similar trend in fluorescence recovery (Figure 6C,D). These results affirm the robustness of Cy3‐Apt_EGFR_@BPNSs for monitoring EGFR dynamics not only in U251 cells but also in U87MG cells.

## 3 Conclusion

Cy3‐Apt_EGFR_@BPNSs serves as a nano‐enabled detector for the quenching and recovery of EGFR and was successfully fabricated via loading a fluorescently tagged EGFR aptamer (Cy3‐Apt_EGFR_) onto BPNSs. Because of the high affinity between the EGFR aptamer and EGFR, Cy3‐Apt_EGFR_@BPNSs accurately identifies EGFR on cancer cells, enabling visualisation through fluorescence labelling. Our research demonstrates that the fluorescence intensity of Cy3‐Apt_EGFR_@BPNSs is directly correlated with EGFR expression levels, providing a more intuitive basis for evaluating these levels. Importantly, we confirmed both the specificity of Cy3‐Apt_EGFR_@BPNSs in detecting EGFR and its capability for the dynamic monitoring of EGFR expression. We believe that this recognition and visualisation detection strategy using Cy3‐Apt_EGFR_@BPNSs represents a promising approach for early tumour diagnosis and therapeutic monitoring. Therefore, people can make immediate medication adjustments based on EGFR expression in the clinic to achieve a better therapeutic effect.

## 4 Experimental Section

### 4.1 Materials

All solvents and chemicals were employed directly as supplied, without any additional purification steps, except where explicitly mentioned. BPNSs were procured from MKNANO (Nanjing, China). The Cy3‐Apt_EGFR_ and Cy3‐R were obtained from Sangon (Shanghai, China). The siRNA sequences were provided by HanYi Biosciences (Guangzhou, China). GelRed and Cell Counting Kit‐8 (CCK‐8) were purchased from Sangon Biotech (Shanghai, China). Hoechst 33342 and DyLight 488 were supplied by Beyotime Biotech (Shanghai, China). PepMute was sourced from Juneng Huida Biotechnology (Wuhan, China). The EGFR antibody, ME1B3 EF660, was provided by Huaqi Sheng Biotechnology (Guangzhou, China). Cell culture media were purchased from Gibco (USA).

### 4.2 Synthesis and Characterisation of Cy3‐Apt_EGFR_@BPNSs

Table S1 presents the sequence information for Cy3‐Apt_EGFR_ and Cy3‐R. In order to avoid re‐agglomeration, BPNSs were treated acoustically for 5 min employing a sonic head with a switching cycle of 5 s on and 2 s off, a 20 Hz frequency, and a 300 W output power in ice water. Cy3‐Apt_EGFR_@BPNSs were formed by mixing Cy3‐Apt_EGFR_ (40 nM) with BPNSs (0.2 mg/mL) in the dark for 5 min. The structure of BPNSs was assessed using TEM (JEM‐2100, Japan). Following this, the size of the BPNSs was determined via AFM (Multimode Nanoscope VIII, Bruker). NanoScope Analysis 2.0 software was employed for statistical assessment of AFM images to quantify the height distribution of BPNSs. The size of the nanoparticles was assessed through DLS (Litesizer). Additionally, a UV–Vis spectrophotometer (UV5Nano model) was used to record the UV–Vis absorption spectra of the samples.

### 4.3 CCK‐8 Assay

NHA cells were inoculated into a 96‐well plate at 3 × 10^4^ (*n* = 3) cells per well. After the cells attached to the plate, the medium was removed and a mixture of DMEM and Cy3‐Apt_EGFR_@BPNSs was added to each well in different proportions, resulting in final concentrations of Cy3‐Apt_EGFR_@BPNSs of 0.2 mg/mL, 0.1 mg/mL, 0.05 mg/mL, 0.025 mg/mL, 0.0125 mg/mL and 0 mg/mL. (The concentration of Cy3‐Apt_EGFR_@BPNSs is determined based on the amount of BPNSs present in the mixed solution) After 6 h of co‐incubation, the CCK‐8 reagent is added and the absorbance of each well is measured at 450 nm using a microplate reader (Biotek, USA).

### 4.4 Stability of Cy3‐Apt_EGFR_@BPNSs

To eliminate unbound Cy3‐Apt_EGFR_, the samples were then subjected to centrifugation at 8000 rpm for 10 min in a 100 KD ultrafiltration. Following this, they were resuspended in PBS and were repeatedly filtered three times on the first, fifth, and ninth day. A UV–Vis spectrophotometer was used to analyse the remaining filtrate, with a range spectrum from 200 nm to 700 nm.

### 4.5 EGFR Knockdown via siRNA

The siRNA sequences were shown in Table S2. One day prior to transfection, cells U251 and U87MG were plated into 6‐well plates, each well containing 7 × 10^5^ cells. Following a 24‐h incubation period, a mixture containing 100 μL PepMute Transfection Buffer, 2.5 μL siRNA and 2.5 μL PepMute was added to 1 mL of medium per well to initiate the transfection process. After a 6‐h co‐incubation, the medium was replaced.

### 4.6 Cell Sublines and Cell Cultures

Cultured at 37°C in a 5% CO_2_ humidified incubator, U251, U87MG and NHA cells (the Institute of Cell Science, Chinese Academy of Sciences) were maintained in DMEM medium consisting of 10% FBS and 1% penicillin–streptomycin.

### 4.7 Fluorescence Assay

BPNSs were co‐incubated with varied concentrations of Cy3‐Apt_EGFR_, whose ratios of the components were adjusted to reach 100 μL in total. The intensity of fluorescence was then measured using a microplate reader (Bio‐Tek, SYNERGY H1, USA) at Ex 520 nm. As for cellular assays, Cy3‐Apt_EGFR_@BPNSs were prepared by mixing BPNSs with Cy3‐Apt_EGFR_ for 5 min (C_Cy3‐AptEGFR_ = 40 nM, C_BPNSs_ = 0.2 mg/mL). This mixture of Cy3‐Apt_EGFR_@BPNSs was subsequently co‐incubated with gradually ascending numbers of the cancer cells (0, 500, 1000, 2000, 5000) in a 96‐well plate for 2 h in the dark. The samples were washed twice in PBS. Fluorescence intensity was assessed in all samples using a standardised method. Four microlitres cells were incubated with a PE‐labelled EGFR antibody at 4°C for 30 min under dark conditions, followed by two rinses with PBS. Following centrifugation, the cells were redispersed in 250 μL of PBS and subjected to fluorescence intensity measurement using a flow cytometer (Beckman Cytoflex, model ZJZXSB‐062). The data were analysed with FlowJo software.

### 4.8 Fluorescence Imaging

5 × 10^5^ U251 and U87MG cells were subsequently plated onto confocal Petri dishes, permitting a 4‐h adhesion period. Following this, Cy3‐Apt_EGFR_@BPNSs was put into the dishes and incubated with the cells for varying durations in the dark at room temperature. After the incubation period, a laser scanning confocal microscope (Nikon AXE NIS‐Elements 5.4, Japan) was used to observe the fluorescence of the cells.

### 4.9 Statistics

Origin 2021 (Origin Laboratories Inc., USA) was utilised for the analysis of all data. Statistical analyses were carried out using a one‐way analysis of variance (ANOVA) for comparisons among multiple groups and a two‐tailed Student's t‐test for comparisons between two groups. All data are reported as mean ± standard deviation (SD), and statistical significance was based on the *p*‐values: ns means no significance, *p < 0.05, **p < 0.01, ***p < 0.001, ****p < 0.0001.

## Author Contributions

X.F., Y.W. and W.Z. made equal contributions to this study. X.F. and Y.Y. conducted the experiments, drafted the manuscript, analysed the data, and executed the statistical analysis. J.Z., X.L. and C.F. offered support and oversight for this research and conducted a critical review of the manuscript. B.L. and Y.L. encompassed the conceptualisation and methodology. W.Z., Y.Z., C.Z. and S.M. participated in the conceptualisation, funding acquisition, methodology, reviewing, writing, supervision and editing.

## Ethics Statement

Ethical approval is not applicable.

## Conflicts of Interest

The authors declare no conflicts of interest.

## Supporting information


**Data S1.** Supporting Information.

## Data Availability

The data that support the findings of this study are available on request from the corresponding author. The data are not publicly available due to privacy or ethical restrictions.
