# Supplementary material for: Nano‐Mediated Fluorescence Switching for Epidermal Growth Factor Receptor Detection
Source: Cell Prolif. 2025 May 14;58(12):e70063. doi: 10.1111/cpr.70063 (PMC12686125; doi:10.1111/cpr.70063)
Supplement: Supplementary file 1 — Data S1. Supporting Information. [file CPR-58-e70063-s001.docx]

**Supplementary Information**

**Nano-Mediated Fluorescence Switching for Epidermal Growth Factor Receptor Detection**

*Xin Fu^1,2,†^* **|** *Yuhao Wang^1,†^* **|** *Wenxin Zhang^3,†,^** **|** *Yuepeng Yang^1^* **|** *Jialin Zeng^1^* **|** *Xiaodie Li^1^* **|** *Chengyu Feng^3^* **|** *Bin Li^2^* **|** *Yingying Liu^4^* **|** *Yinan Zhang^5,^** **|** *Chao Zhang^1,2,^** **|** *Sicong Ma^6,7,^**

^1^Department of Oncology, Zhujiang Hospital, Southern Medical University, Guangzhou 510282, P.R. China.

^2^School of Inspection, Ningxia Medical University, Yinchuan 750004, P.R. China.

^3^Department of Pediatric Hematology, Zhujiang Hospital, Southern Medical University, Guangzhou 510282, P.R. China.

^4^School of Chemistry and Chemical Engineering, Center for Transformative Molecules, Zhangjiang Institute for Advanced Study and National Center for Translational Medicine (Shanghai), Shanghai Jiao Tong University, Shanghai 200240, P.R. China.

^5^School of Chemical Science and Engineering, Tongji University, Shanghai 200092, P.R. China.

^6^Department of Intensive Care Medicine, Zhujiang Hospital, Southern Medical University, Guangzhou 510282, P.R. China.

^7^Global Health Research Center, Guangdong Provincial People’s Hospital, Guangdong Academy of Medical Sciences, Guangzhou 510280, P.R. China.

*^†^*Xin Fu, Yuhao Wang and Wenxin Zhang made equal contributions to this study.

*Correspondence: Wenxin Zhang (zwxsakura@gmail.com) **|** Yinan Zhang (yinan_zhang@tongji.edu.cn) **|** Chao Zhang (czhangsinap@163.com) **|** Sicong Ma (masc21@163.com)


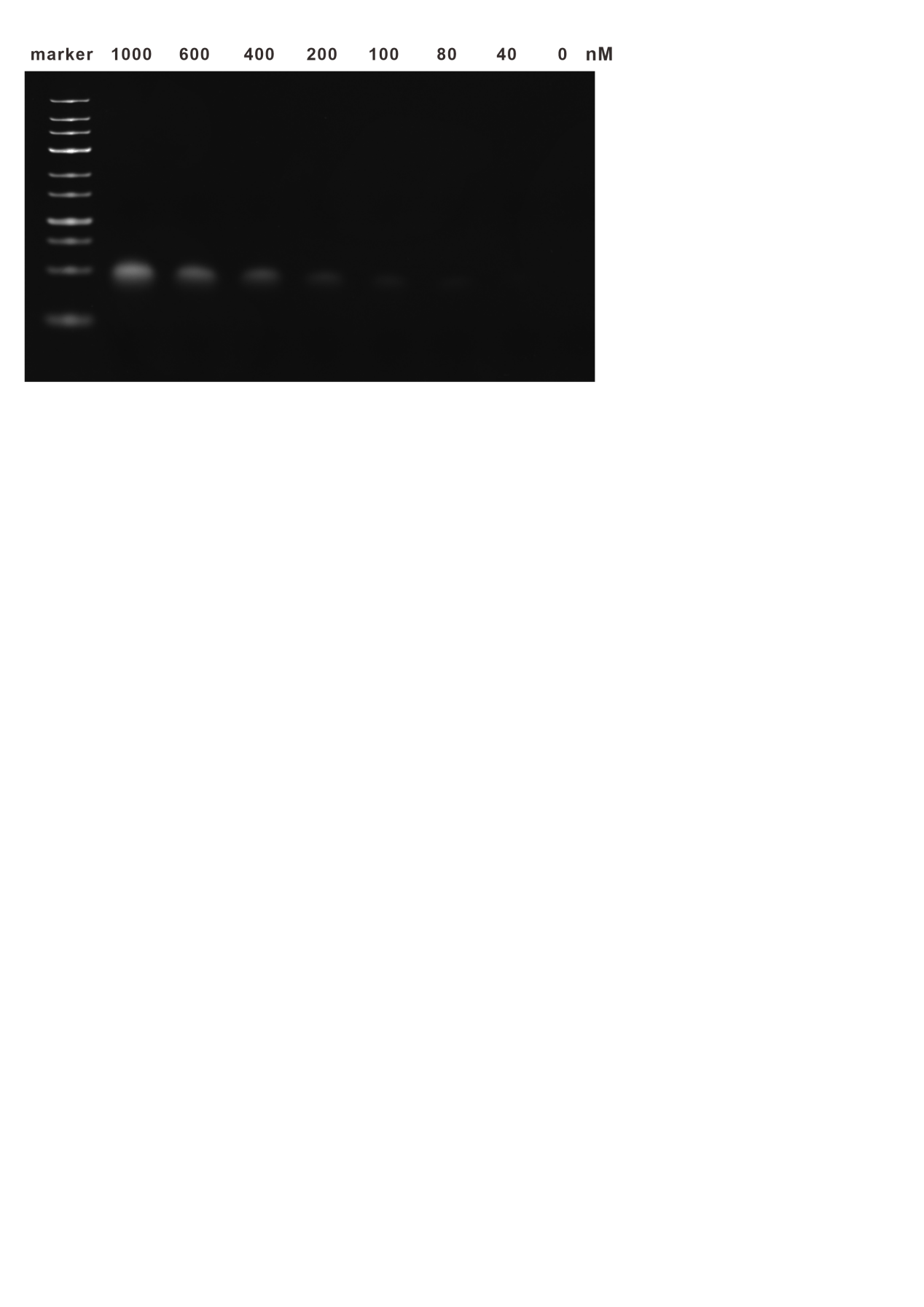


**Figure S1.** Optimised ratio of EGFR aptamer to BPNSs investigated by gel retardation quantification: from left to right, Apt concentrations were 1000 nM, 600 nM, 400 nM, 200 nM, 100 nM, 80 nM, 40 nM and 0 nM corresponding to the same BPNSs concentration.


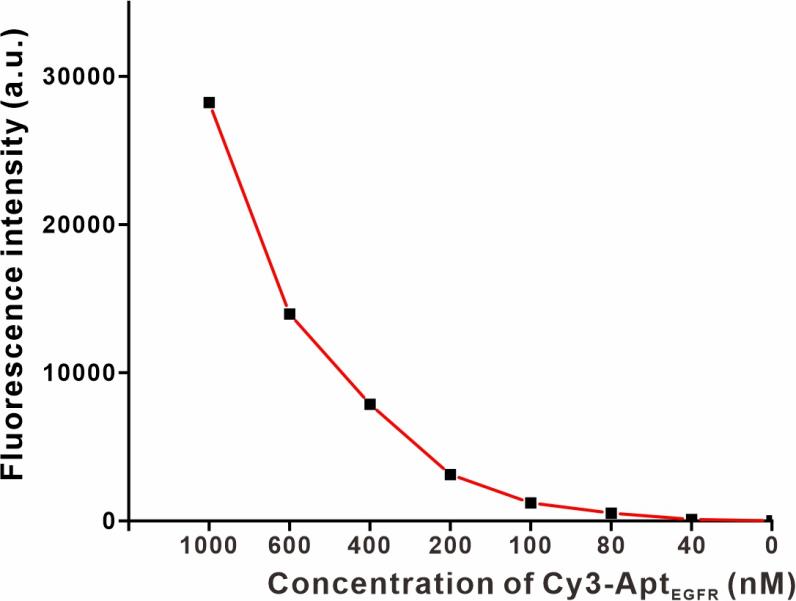


**Figure S2.** Fluorescence quantitative analysis of gel imaging, corresponding to Figure S1.


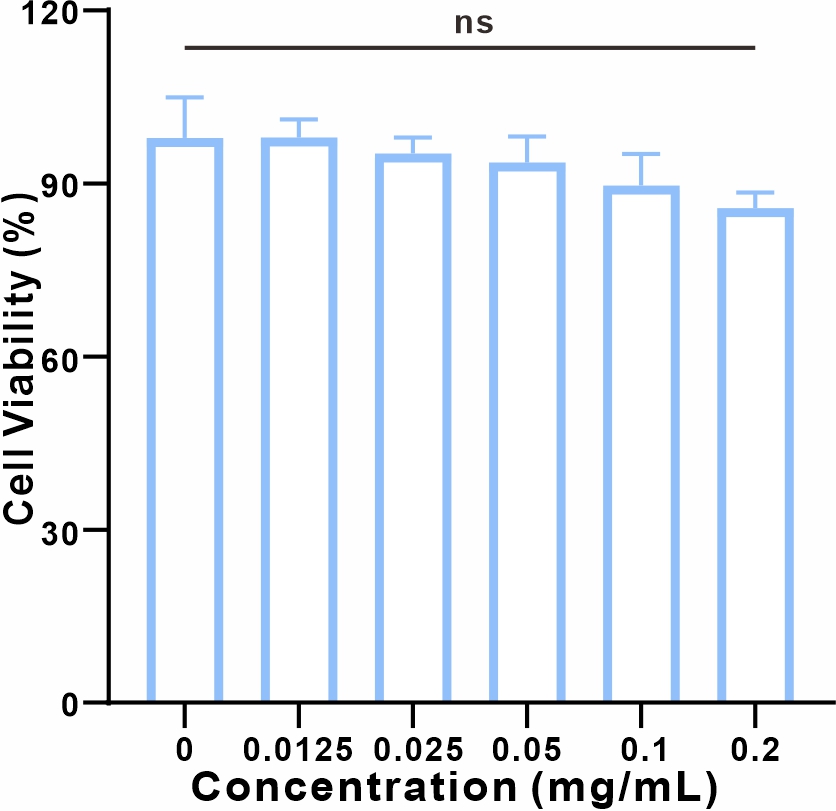


**Figure S3.** Cell viability of NHA cells after treatment with Cy3-Apt_EGFR_@BPNSs. Data are presented as the mean ± SD (n=3, ns means no significance).


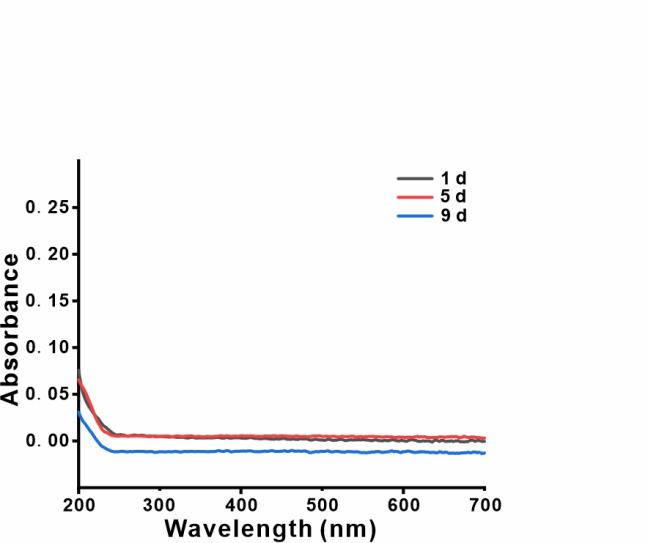


**Figure S4.** UV absorbance spectra of Cy3-Apt_EGFR_@BPNSs filtrates at day 1, 5 and 9 at room temperature.


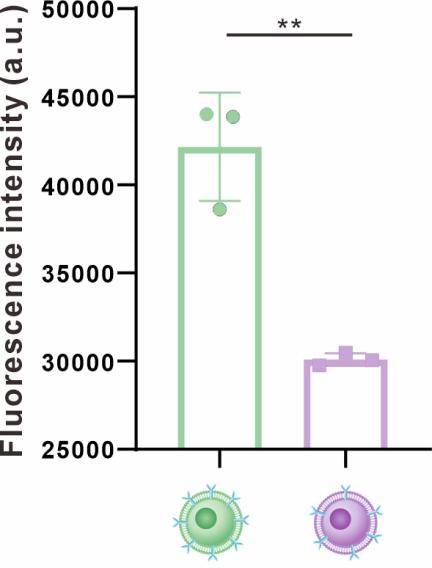


**Figure S5.** Statistical analysis of EGFR expression between U251 and U87MG cell lines, corresponding to Fig. 2B and 3B. ***p*<0.01.


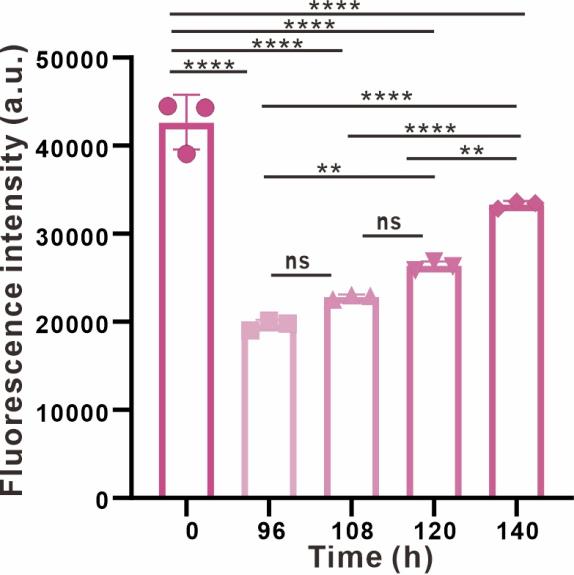


**Figure S6.** The statistical analysis corresponding to the flow cytometry results in Fig. 5B. ns means no significance, ***p*<0.01, *****p*<0.0001.


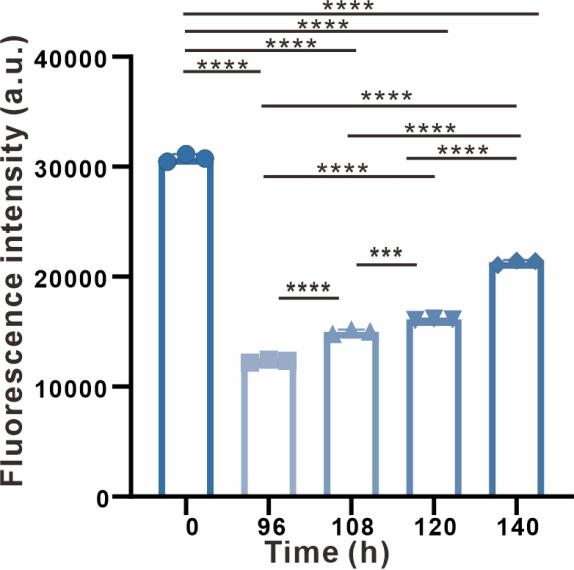


**Figure S7.** The statistical analysis corresponding to the flow cytometry results in Fig. 6B. ****p*<0.001, *****p*<0.0001.

**Table S1.** Cy3-Apt_EGFR_ and Cy3-R sequences.

| Type | Sequences (5’-3’) |
| --- | --- |
| Cy3-Apt_EGFR_ | Cy3-GCCUUAGUAACGUGCUUUGAUGUCGAUUCGACAGGAGGC |
| Cy3-R | Cy3-UUCGUACCGGGUAGGUUGGCUUGCACAUAGAACGUGUCA |

**Table S2.** The siRNA sequences.

| siRNA 1 | UCCAGAGGAUGUUCAAUAATTUUAUUGAACAUCCUCUGGATT |
| --- | --- |
| siRNA 2 | CACAAAGCAGUGAAUUUAUTTAUAAAUUCACUGCUUUGUGTT |
| siRNA 3 | UGCUCUGAAAUCUCCUUUATTUAAAGGAGAUUUCAGAGCATT |
